# Associations of depressive symptoms with lower extremity function and balance in Korean older adults

**DOI:** 10.4178/epih.e2024021

**Published:** 2024-01-15

**Authors:** Bong Jo Kim, Kyupin Ha, Hyun Soo Kim, Hye Ran Bae, Minkook Son

**Affiliations:** 1 Department of Physiology, Dong-A University College of Medicine, Busan, Korea; 2 Department of Psychiatry, Dong-A University College of Medicine, Busan, Korea; 3 Department of Data Sciences Convergence, Dong-A University Interdisciplinary Program, Busan, Korea

**Keywords:** Depression, Lower extremity, Postural balance, Aged

## Abstract

**OBJECTIVES:**

The relationship of depressive symptoms to lower extremity function and balance, especially in older adults without a depression diagnosis, remains unclear. Therefore, our study analyzed this relationship using a large sample of Korean older adults.

**METHODS:**

We used data from the Korean National Health Insurance Service’s Health Screening Program database. Individuals aged 66 years who had undergone the National Screening Program for Transitional Ages in Korea and were without a diagnosis of depressive disorder were included. The lower extremity function and balance were evaluated using 2 physical tests, while depressive symptoms were assessed using a 3-question survey. Multivariable-adjusted logistic regression analysis was used to examine the association between depressive symptoms and lower extremity function and balance.

**RESULTS:**

Among 66,041 individuals, those with depressive symptoms showed significantly higher rates of abnormal lower extremity function and abnormal balance. The adjusted odds ratios (aORs) and 95% confidence intervals (CIs) for the association of depressive symptoms to abnormal lower extremity function and abnormal balance were (aOR, 1.34; 95% CI, 1.25 to 1.44) and (aOR, 1.38; 95% CI, 1.29 to 1.48), respectively. Assessment of the relationship based on depressive symptom scores revealed that higher scores were associated with higher aORs (p for trend <0.001). Subgroup analyses further confirmed this relationship, especially among patients with cerebrovascular disease or dementia.

**CONCLUSIONS:**

This study revealed an association between depressive symptoms and the abnormal lower extremity function and balance of 66-year-old individuals without a diagnosis of depressive disorder.

## GRAPHICAL ABSTRACT

[Fig f3-epih-46-e2024021]

## Key Message

• This study investigated the associations of depressive symptoms with lower extremity function and balance in 66-year-old individuals without a diagnosis of major depressive disorder.

• Older adults with depressive symptoms showed significantly higher levels of abnormal lower extremity function and balance than those without.

• Higher depressive symptom scores were associated with higher odds ratios for abnormal lower extremity function and balance.

## INTRODUCTION

Depression in older adults has become an increasingly important issue. It can negatively impact health and lead to social isolation, thus decreasing daily activities and quality of life [1]. Depressive symptoms are characterized by diminished overall mental function (thinking, motivation, interest, behavior, and sleep), decreased physical activity, and negative mood [2]. However, the presence of depressive symptoms does not receive the same level of attention as a major depression diagnosis [2].

Lower extremity and balance functions are essential aspects of daily life, requiring the integration of proprioceptive sensory information to generate appropriate motor responses [3,4]. Age-related loss of lower extremity function and balance increases the risk of falling, which can result in serious injury [5]. Over one-third of community-dwelling older adults experience at least 1 fall per year, with 10-15% resulting in serious injury [6-9]. Moreover, these falls result in significant societal and economic costs, with healthcare costs increasing as the proportion of older adults in the population increases [2,3]. Therefore, elucidating the factors associated with lower extremity function and balance is crucial for predicting and preventing falls in older adults as well as reducing the economic burden on individuals and society.

Previous studies have demonstrated a relationship between depression and lower extremity function and balance. Wang et al. [10] and Zhang et al. [11] reported a correlation between depression and gait. Murri et al. [12] found that depression can cause impairments in gait, balance, and posture, and that antidepressants and exercise can improve the motor function characteristics of depression. These findings suggest that depressive symptoms may be early indicators of impaired lower extremity function and balance [13-15]. Although several studies have investigated the relationship between depression and lower extremity function and balance, there is limited research on this association in older adults. In addition, since geriatric depression is often underdiagnosed and undertreated [16], there is a paucity of large-scale studies on the association between depressive symptoms and the lower extremity function and balance of older adults without a diagnosis of depressive disorder. Therefore, our study analyzed this association in a large sample of older adults (aged 66 years only) in Korea using the National Health Insurance Service Health Screening (NHIS-HEALS) database, which includes data obtained in the National Screening Program for Transitional Ages in Korea.

## MATERIALS AND METHODS

### Data source

The data used in this study were sourced from the NHIS-HEALS database [17]. The NHIS database contains information on 98% of the Korean population and includes data regarding their insurance claims. The NHIS-HEALS database contains information on 514,794 individuals randomly selected from the 5.1 million participants in the national health check-up program conducted between January 2002 and December 2003. The selected individuals were aged 40 years to 79 years by the end of December 2003. The NHIS-HEALS database contains demographic and socioeconomic information, medical record information regarding healthcare usage, and health screening information with blood test results. In Korea, when an individual reaches the age of 66 years, they also undergo the National Screening Program for Transitional Ages, which includes a survey regarding depressive symptoms [18]. Additional information regarding this database can be found on the relevant website (https://nhiss.nhis.or.kr/bd/ab/bdaba000eng.do).

### Study population

**Figure 1.**
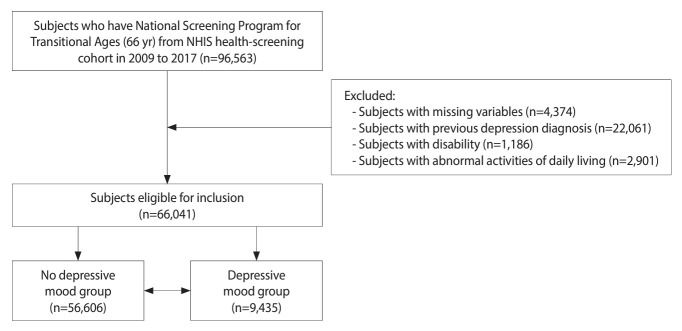
Sampling flowchart used to study the association between depressive symptoms and lower extremity and balance function in older adults. NHIS, National Health Insurance Service.

This study considered recent depressive symptoms in 66-year-old individuals, as recorded in the National Screening Program for Transitional Ages in Korea. Three questions were used to assess depressive symptoms: (1) “Have you experienced a significant loss in activity or motivation?”, (2) “Do you perceive yourself as useless in your current state?”, and (3) “Do you feel hopeless about your current situation?”. Among the 96,563 participants in the National Screening Program for Transitional Ages in Korea between 2009 and 2017, we excluded those with missing values (n = 4,374) or a previous diagnosis of depression based on previous insurance claims containing the corresponding International Classification of Diseases-10th revision (ICD-10) codes (n = 22,061). Based on questionnaire answers, we excluded individuals with disabilities such as lower extremity dysfunction (n = 1,186) or abnormal activities of daily living (ADLs; n = 2,901). Lower extremity dysfunction and walking disabilities were determined based on an examiner’s assessment. The corresponding variables resulting from this determination were available in the NHIS-HEALS database. The ADLs assessment involved 6 categories, including self-washing, self-dressing, self-eating, self-voiding, self-food preparation, and self-transportation. Ultimately, 66,041 participants were included in this study, and those participants with affirmative responses to any questions about recent depressive symptoms were advised to seek psychological counseling. Based on this recommendation, we classified 56,606 and 9,435 individuals as having or lacking depressive symptoms, respectively. A flowchart of the study population is shown in Figure 1.

### Covariates

The socio-demographic characteristics of the study sample, including sex and income level, were included as covariates. The income levels were divided into quartile groups. Body mass index (BMI), waist circumference, systolic blood pressure, and diastolic blood pressure; fasting blood glucose, total cholesterol, triglyceride, high-density lipoprotein (HDL)-cholesterol, low-density lipoprotein (LDL)-cholesterol, and hemoglobin levels; and glomerular filtration rate (GFR) were extracted from the health screening data. The GFR was calculated using the Modification of Diet in Renal Disease equation [19]. Based on health screening and prescription data, metabolic syndrome was included as a covariate. Metabolic syndrome was defined as the presence of 3 or more of the following 5 criteria: (1) waist circumference ≥ 90 cm in male or ≥ 85 cm in female, (2) systolic blood pressure ≥ 130 mmHg and/or diastolic blood pressure ≥ 85 mmHg or the use of antihypertensive drugs, (3) triglyceride level ≥ 150 mg/dL or the use of dyslipidemia drugs, (4) HDL-cholesterol level < 40 mg/dL in male and < 50 mg/dL in female or the use of dyslipidemia drugs, and (5) fasting blood glucose ≥ 100 mg/dL or the use of antidiabetic drugs. Moreover, we included underlying diseases that could affect physical function as covariates. Based on ICD-10 codes, these included cerebrovascular disease, ischemic heart disease, chronic obstructive pulmonary disease, and dementia [20-23].

Information regarding smoking habits, alcohol consumption, and regular exercise status was obtained through questionnaires. Based on their smoking habits, patients were classified as non-smokers, ex-smokers, or smokers. Patients were classified according to their drinking status as non-drinkers or drinkers. Regular exercise was defined as at least 5 exercise sessions per week.

### Definition of abnormal lower extremity function and balance

Two physical function tests were performed to evaluate lower extremity function and balance in older adults. Lower extremity function was assessed using a 3-meter timed up-and-go test, and times were recorded. Balance was assessed using a single-leg stance test, and times were recorded. According to NHIS criteria, lower extremity function was normal if the duration was < 10 seconds, while balance function was normal if the duration was > 6 seconds or > 10 seconds with eyes closed or open, respectively.

### Statistical analysis

Data provided by the NHIS were analyzed using R version 4.3.0 (R Foundation for Statistical Computing, Vienna, Austria) and SAS version 9.4 (SAS Institute Inc., Cary, NC, USA). Baseline characteristics are presented as means with standard deviations for continuous variables or numbers with percentages for categorical variables. Between-group comparisons of continuous and categorical variables were performed using Student t-tests and chi-square tests, respectively. Multivariable-adjusted logistic regression analysis was conducted to confirm the association between depressive symptoms and lower extremity function and balance. The results are presented as odds ratios (ORs) and 95% confidence intervals (CIs). The model was adjusted for multiple covariates, including sex, income level, BMI, hemoglobin level, GFR, smoking habits, alcohol consumption, regular exercise status, metabolic syndrome, cerebrovascular disease, ischemic heart disease, chronic obstructive pulmonary disease, and dementia. Statistical significance was set at p-value < 0.05.

### Ethics statement

The study protocol was approved by the Institutional Review Board of Dong-A University (2-1040709-AB-N-01-202305-HR-027-02). The need to obtain informed consent from the participants was waived because the NHIS-HEALS data were provided anonymously and strictly complied with confidentiality regulations.

## RESULTS

### Baseline characteristics of the study population according to the presence of depressive symptoms

The baseline characteristics of the participants according to the presence of depressive symptoms are presented in Table 1. There was no between-group difference in age. However, the proportion of females was higher in the group with depressive symptoms than in the group without depressive symptoms. Compared with the group without depressive symptoms, the group with depressive symptoms exhibited lower blood pressures, hemoglobin levels, and GFRs, as well as higher total and LDL-cholesterol levels. Furthermore, the proportion of current smokers was higher in the group with depressive symptoms than in the group without depressive symptoms, whereas that of alcohol drinkers and regular exercisers was lower. All underlying diseases were more prevalent in the group with depressive symptoms than in the group without depressive symptoms. Abnormal lower extremity function and balance were significantly higher in the group with depressive symptoms than in the group without depressive symptoms.

### Association between depressive symptoms and abnormal lower extremity function

The association between depressive symptoms and abnormal lower extremity function is shown in Table 2. There were 4,404 (7.8%) and 1,005 (10.7%) patients with abnormal lower extremity function in the groups without depressive symptoms and with depressive symptoms, respectively. The crude OR for depressive symptoms and abnormal lower extremity function was 1.41 (95% CI, 1.32 to 1.52). The adjusted OR was 1.34 (95% CI, 1.25 to 1.44). When the 3 questions related to depressive symptoms were scored and summed, the strength of the association increased as the score increased, with the highest score group having an adjusted OR of 1.57 (95% CI, 1.37 to 1.80) relative to the lowest score group (p for trend < 0.001). Sub-analysis of the questions revealed that all 3 items were significantly associated with abnormal lower extremity function, with the question “Do you feel hopeless about your current situation?” showing the strongest association (adjusted OR, 1.47; 95% CI, 1.33 to 1.64).

### Association between depressive symptoms and abnormal balance

The association between depressive symptoms and abnormal balance is shown in Table 2. There were 1,127 (11.9%) and 4,790 (8.5%) patients with abnormal balance in the groups with and without depressive symptoms, respectively. The crude OR for depressive symptoms and abnormal balance was 1.47 (95% CI, 1.37 to 1.57), with an adjusted OR of 1.38 (95% CI, 1.29 to 1.48). When the 3 questions related to depressive symptoms were scored and summed, the strength of the association increased as the scores increased (p for trend < 0.001). Sub-analysis of the questions revealed that they were all significantly linked to abnormal balance, with the question “Do you perceive yourself as useless in your current state?” having the strongest association (adjusted OR, 1.46; 95% CI, 1.31 to 1.63).

### Subgroup analysis

**Figure 2.**
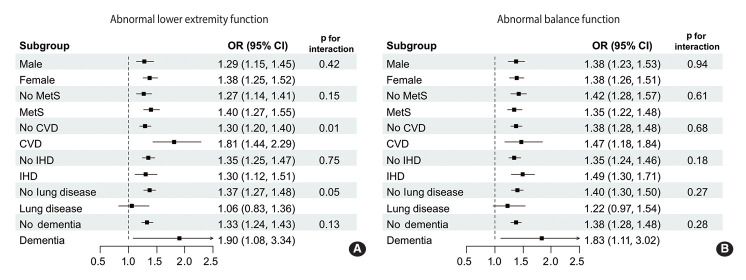
Subgroup analysis of the association between depressive symptoms and (A) abnormal lower extremity function and (B) balance. OR, odds ratio; CI, confidence interval; Mets, metabolic syndrome; CVD, cerebrovascular disease; IHD, ischemic heart disease.

Subgroup analysis of the association between depressive symptoms and abnormal functions is shown in Figure 2. Notably, the association was particularly strong among participants with cerebrovascular disease or dementia.

## DISCUSSION

In this study, we utilized nationwide data to investigate the association between depressive symptoms and the lower extremity function and balance of 66-year-old individuals not diagnosed with a major depressive disorder. Overall, the group with depressive symptoms showed significantly higher levels of abnormal lower extremity and balance function than the group without depressive symptoms. Although there were between-group differences in the baseline characteristics, multivariable-adjusted logistic regression analysis confirmed the association between depressive symptoms and lower extremity function and balance. When evaluating the association based on depressive symptom scores, higher scores were associated with higher ORs for both abnormal lower extremity function and abnormal balance.

Notably, among the 3 assessment questions, abnormal lower extremity function was strongly associated with question 3 (related to hopelessness), while abnormal balance function was strongly associated with question 2 (related to uselessness). These results suggest that different depressive symptoms may be related to different types of muscle function. Considering the variety of depressive symptoms and types of depression [24], additional research is warranted to confirm these findings.

Previous studies have shown a significant association between reduced depressive symptoms and better muscle strength and balance as well as higher walking speed [25,26]. In addition, depressive symptoms have been positively correlated with impairments in lower extremity function, especially among patients with peripheral artery disease [27,28]. Previous studies have also found impaired lower extremity function and balance among individuals diagnosed with depression [29]. Taken together, these findings suggest that depressive disorders and symptoms are associated with postural abnormalities during standing that may increase the risk of falls.

Individuals diagnosed with depression have been shown to experience physical decline [29-31], with several studies proposing biologically mediated pathways and psychological mechanisms underlying this relationship. Animal and human studies have demonstrated that psychological distress can lead to neural, hormonal, and immunological changes. Depressive symptoms may increase vulnerability to disease and negatively impact physical health by elevating sympathetic tone, reducing vagal tone, and inducing immunosuppression [32,33]. Furthermore, persistent somatic symptoms of depression, including fatigue or sleeplessness, can deteriorate an individual’s health over time. Regarding psychological mechanisms, depressive symptoms may discourage individuals from seeking adequate medical attention and rehabilitation, adhering to treatment regimens, or adopting healthy lifestyle habits like exercising, abstaining from smoking, and maintaining nutritious eating habits.

Abnormal lower extremity function and balance have been associated with multiple factors, including older age, underlying disease, and psychosocial factors [30,34,35]. Therefore, our findings suggest that special attention should be paid to people with abnormal lower extremity function and balance. The study by Mokhtari et al. [36] concluded that Pilates can be effective in reducing depression and improving both dynamic and static balance. It is essential that individuals who may develop depression be promptly identified and provided with appropriate interventions.

This study had several limitations. First, because we analyzed health screening data from the National Screening Program for Transitional Ages in the NHIS-HEALS database, we only targeted 66-year-old Koreans. Further studies that generalize our findings to other age groups, are warranted. By expanding the target age group for screening, individuals who may develop depression can be promptly detected and receive appropriate interventions. Second, the cross-sectional study design did not allow us to establish a causal relationship; thus, further research employing different study designs and methods is required to ascertain causality. According to Dauwan et al. [37], exercise has a positive impact on depressive symptoms and enhances various cognitive domains. Therefore, further studies are warranted to explore the effects of different exercise types on both physical function and depressive symptoms [37]. Third, hidden confounding factors could not be extracted from the NHIS-HEALS database. Considering these limitations, it is important to interpret the results of this study with caution.

Despite these limitations, however, this study has several strengths. It was the first nationwide study, using the largest claims dataset in Korea that included health screening data from detailed lifestyle questionnaires, laboratory results, and anthropometric measurements. Second, our various analyses consistently showed the association between depressive symptoms and abnormal lower extremity function and balance.

In conclusion, we found a significant association between depressive symptoms and abnormal lower extremity function and balance in 66-year-old adults who used screening questionnaires to report their depressive symptoms. These findings may contribute to understanding the factors that influence the health status and functioning of older adults affected by mood disturbances. Moreover, they may inform the development of strategies aimed at preventing and treating depression, specifically in older adults. Further research is warranted using diverse study designs and methodologies to establish causality.

## Figures and Tables

**Figure f3-epih-46-e2024021:**
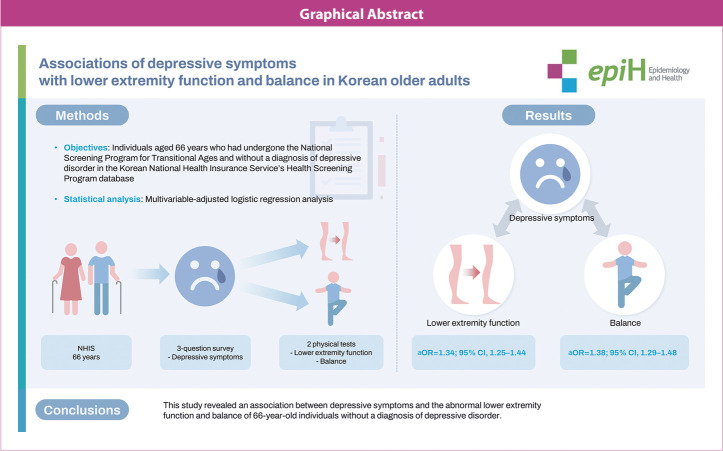


**Table 1.** Baseline characteristics of the Korean population aged 66 years according to the presence or absence of depressive symptoms (n=66,041)

Characteristics	Depressive symptoms		p-value
	Without (n=56,606)	With (n=9,435)	
Demographics			
Sex			<0.01
Male	32,131 (56.8)	4,366 (46.3)	
Female	24,475 (43.2)	5,069 (53.7)	
Income level (quartile)			0.01
1st	10,307 (18.2)	1,704 (18.1)	
2nd	12,489 (22.1)	1,948 (20.6)	
3rd	17,219 (30.4)	2,968 (31.5)	
4th	16,591 (29.3)	2,815 (29.8)	
Health screening			
Body mass index (kg/m^2^)	24.2±2.9	24.2±3.1	0.99
Waist circumflex (cm)	83.2±8.1	83.1±8.4	0.26
Systolic blood pressure (mmHg)	127.8±14.7	127.1±15.0	<0.01
Diastolic blood pressure (mmHg)	77.5±9.4	77.0±9.7	<0.01
Fasting blood glucose (mg/dL)	104.1±25.2	104.1±26.1	0.99
Total cholesterol (mg/dL)	194.4±38.2	195.7±43.4	<0.01
Triglyceride (mg/dL)	130.1±75.5	131.4±76.1	0.12
HDL-cholesterol (mg/dL)	54.0±18.8	53.7±15.5	0.14
LDL-cholesterol (mg/dL)	114.7±35.6	115.8±40.1	0.01
Hemoglobin (g/dL)	13.9±1.4	13.7±1.5	<0.01
Glomerular filtration rate (mL/min/1.73 m^2^)	80.3±34.8	79.4±25.6	0.02
Current smoker	6,592 (11.6)	1,274 (13.5)	<0.01
Alcohol drinking	17,579 (31.1)	2,752 (29.2)	<0.01
Regular exercise	5,528 (9.8)	645 (6.8)	<0.01
Underlying disease			
Metabolic syndrome	26,095 (46.1)	4,596 (48.7)	<0.01
Cerebrovascular disease	3,594 (6.3)	803 (8.5)	<0.01
Ischemic heart disease	12,000 (21.2)	2,341 (24.8)	<0.01
Chronic obstructive pulmonary disease	4,363 (7.7)	887 (9.4)	<0.01
Dementia	548 (1.0)	143 (1.5)	<0.01
Lower extremity function			
Timed up-and-go test (sec)	8.0±2.3	8.2±2.5	<0.01
Abnormal	4,404 (7.8)	1,005 (10.7)	<0.01
Balance function			
Single-leg stance test (sec)	19.0±9.2	17.7±9.3	<0.01
Abnormal	4,790 (8.5)	1,127 (11.9)	<0.01

Values are presented as number (%) or mean±standard deviation. HDL, high-density lipoprotein; LDL, low-density lipoprotein.

**Table 2.** Association between depressive symptoms and abnormal lower extremity function and balance in 66-year-old Korean adults (n=66,041)

Participants	Events	Crude	p-value	Adjusted^1^	p-value
Abnormal lower extremity function					
Depressive symptoms					
Without (n=56,606)	4,404	1.00 (reference)		1.00 (reference)	
With (n=9,435)	1,005	1.41 (1.32, 1.52)	<0.001	1.34 (1.25, 1.44)	<0.001
Sum of depressive symptom responses					
0 (n=56,606)	4,404	1.00 (reference)		1.00 (reference)	
1 (n=6,012)	589	1.29 (1.18, 1.41)	<0.001	1.23 (1.12, 1.35)	<0.001
2 (n=1,337)	160	1.61 (1.36, 1.91)	<0.001	1.50 (1.26, 1.77)	<0.001
3 (n=2,086)	256	1.66 (1.45, 1.90)	<0.001	1.57 (1.37, 1.80)	<0.001
Question 1. Have you experienced a significant loss in activity or motivation?					
No (n=57,912)	4,541	1.00 (reference)		1.00 (reference)	
Yes (n=8,129)	868	1.41 (1.30, 1.52)	<0.001	1.34 (1.24, 1.44)	<0.001
Question 2. Do you perceive yourself as useless in your current state?					
No (n=62,794)	5,024	1.00 (reference)		1.00 (reference)	
Yes (n=3,247)	385	1.55 (1.39, 1.73)	<0.001	1.46 (1.31, 1.63)	<0.001
Question 3. Do you feel hopeless about your current situation?					
No (n=62,473)	4,985	1.00 (reference)		1.00 (reference)	
Yes (n=3,568)	424	1.56 (1.40, 1.73)	<0.001	1.47 (1.33, 1.64)	<0.001
Abnormal balance function					
Depressive symptoms					
Without (n=56,606)	4,790	1.00 (reference)		1.00 (reference)	
With (n=9,435)	1,127	1.47 (1.37, 1.57)	<0.001	1.38 (1.29, 1.48)	<0.001
Sum of responses for depressive symptoms					
0 (n=56,606)	4,790	1.00 (reference)		1.00 (reference)	
1 (n=6,012)	685	1.39 (1.28, 1.51)	<0.001	1.32 (1.21, 1.44)	<0.001
2 (n=1,337)	174	1.62 (1.38, 1.90)	<0.001	1.48 (1.26, 1.75)	<0.001
3 (n=2,086)	268	1.60 (1.40, 1.82)	<0.001	1.51 (1.32, 1.72)	<0.001
Question 1. Have you experienced a significant loss in activity or motivation?					
No (n=57,912)	4,944	1.00 (reference)		1.00 (reference)	
Yes (n=8,129)	973	1.46 (1.35, 1.57)	<0.001	1.38 (1.28, 1.48)	<0.001
Question 2. Do you perceive yourself as useless in your current state?					
No (n=62,794)	5,496	1.00 (reference)		1.00 (reference)	
Yes (n=3,247)	421	1.55 (1.40, 1.73)	<0.001	1.46 (1.31, 1.63)	<0.001
Question 3. Do you feel hopeless about your current situation?					
No (n=62,473)	5,474	1.00 (reference)		1.00 (reference)	
Yes (n=3,568)	443	1.48 (1.33, 1.64)	<0.001	1.39 (1.25, 1.54)	<0.001

Values are presented as odds ratio (95% confidence interval).

1 Adjusted for sex, income level, body mass index, hemoglobin level, glomerular filtration rate, smoking status, alcohol consumption, regular exercise status, and presence of metabolic syndrome, ischemic heart disease, cerebrovascular disease, lung disease, or dementia.
